# Throwing like a girl: a critique of past approaches and an illustrated proposal for a path forward

**DOI:** 10.1186/s13293-025-00759-8

**Published:** 2025-10-16

**Authors:** Anne Fausto-Sterling

**Affiliations:** Department of Molecular Biology, Cell Biology and Biochemistry. Providence, Brown University, Rhode Island, 02912 USA

**Keywords:** Phenomenology, Developmental process, Infant motor development, Throwing like a girl, Developmental dynamics, Developmental phenomenology, Gender/sex

## Abstract

**Background:**

The phrase “Throwing like a girl” persists in popular culture and in scientific research as a trope about biological differences between males and females. In this review/theoretical paper I critically examine the support for the idea that sex differences in throwing style and force result from innate biological difference.

**Methods:**

This article contains (1) a limited critical review of selected literature, (2) the application of a systems approach to the development of ball play that starts the study of throwing capacity as it develops in infancy and considers its emergence going forward and (3) a demonstration of this approach using qualitative descriptions of events (at ages 9–15 months) involving toddlers’ first engagements in ball play.

**Results:**

The literature cited to support the claim that sex differences in throwing are a ubiquitous/universal feature of human children is weak. When I compared two toddlers over several months, starting at the time of their first ball throwing game, I learned that the boy and his mother played frequently even before he could walk. In contrast, the girl began ball play at an older age that coincided with her learning to walk. For 8 additional children, the boys started playing ball almost 2.5 months earlier than the girls, and all before they could walk.

**Conclusions:**

The boy could raise the ball higher and throw it further at 13–14 months because he had practiced more from the more stable sitting and kneeling positions which allowed him to master double-handed overhead ball throwing before he could walk. The girl tried throwing the ball while walking unsteadily. Thus, when trying to raise the ball above her head, she often fell, and could not throw it very far. I conclude that to understand sex differences in embodied motor skills in children requires that we study the processes of motor learning beginning at birth.

## Abstract (plain language summary)

The belief that boys and girls throw differently is widespread. The scientific findings supporting this belief, while numerous, vary greatly in terms of method and the age of the children studied. Furthermore, research projects are usually based on a two-fold binary paradigm: first that the important contrasts are between boys and girls; second that studies of variation in throwing form illuminate the degree to which nature compared to nurture contribute to reported differences. Here, I present an alternate paradigm—the analysis of motor skills as they first develop during infancy. This paradigm embraces nurture/nature and gender/sex as entangled and best understood as a dynamic whole; it also allows us to understand throwing form and skill as a continuum or spectrum rather than a binary construct. To offer a concrete instance, I describe the ball-playing activities of 10 infants aged 6–15 months and their mothers. The five boys all began to play active ball games with their mothers at the average age of 9.7 months. This early playing happened before they could walk, and they mastered stable two-handed overhead throwing from a sitting position. The girls and their mothers did not play ball until the babies averaged 12.1 months. Four out of five of the girls were learning to walk and throw at the same time, a condition of instability that made it difficult for them to fully raise the ball above their head without falling. We hypothesize that differences in timing and frequency of play events lead to continuous variability in the embodiment of throwing skills.

## Background

Taunts can hurt– even for battle-hardened adults. I was about sixty-five, and attending an annual gathering with a group of long-time friends. I had been outside happily playing catch with my dog, and was feeling pretty good about my physical self (not a bad throwing arm for an old lady) when one of our party started to make fun of me. I had, he teased, been throwing like a girl. Not only did this hurt my feelings then, but even now, over a dozen years later, the incident stings. Throwing like a girl? What kind of indictment was this? If there really is such a thing, how does it develop? In this paper I will combine a brief history of the epithet with some preliminary data gathered on individual and dyadic interactions in infancy to challenge researchers to think about gender/sex differentiation using developmental systems analyses. Throughout this article I rely on the term gender/sex to indicate the entanglement of the body with the culture within which it exists [1–3].

In 1980 feminist philosopher Iris Young wrote an essay entitled “Throwing like a Girl: A Phenomenology of Feminine Body Comportment Motility and Spatiality” [4]. Young reached back to an odd, unsupported comment in a classic essay by Erwin Straus, a phenomenologist and neurologist who pioneered a wholistic approach to medicine and psychiatry. In “An Upright Posture” Straus speculated on the psychological meanings of uprightness, and, toward the end of his otherwise gender-neutral piece, inserted two paragraphs on “the remarkable difference in the manner of throwing of the two sexes.” [5] (pp. 552–553).

According to Straus, girls held most of their body still while throwing only with their partially extended arm. Boys put their entire body into the throw, enabling greater thrust and accuracy. Taking Straus’ description at face value, Young agreed that this difference represented masculine and feminine ways of moving the body through space. She defined femininity as “a set of structures and conditions which delimit the typical *situation* of being a woman in a particular society…” (p.140, italics in original). Her analysis was phenomenological—a consideration of the lived body described by Merleau-Ponty, and feminist, based on the existentialist views of Simone de Beauvoir [4].

When girls and women attempt an overhand ball toss, according to Young, they use specific behaviors and movement styles through space. These do not involve anatomy or physiology or some “mysterious feminine ‘essence’” (p. 152) Instead, throwing like a girl was shaped by sexist oppression in contemporary society. “Women in sexist society”, she wrote, “are physically handicapped…”. (p. 152) Girls’ play is ”more sedentary and enclosing”; they have fewer chances to develop the spatial skills needed for accurate throwing. They learn to move like a girl, “walking…tilting their head…standing and sitting…gesturing…like a girl”. (p. 153). Girls also learn to live in a body that is an object, not a subject. “She gazes at it in the mirror, worries about how it looks to others, prunes it, shapes it, molds and decorates it.” (p.154). For Young, throwing like a girl was one “natural” outcome of these behaviors.

**Fig. 1:**
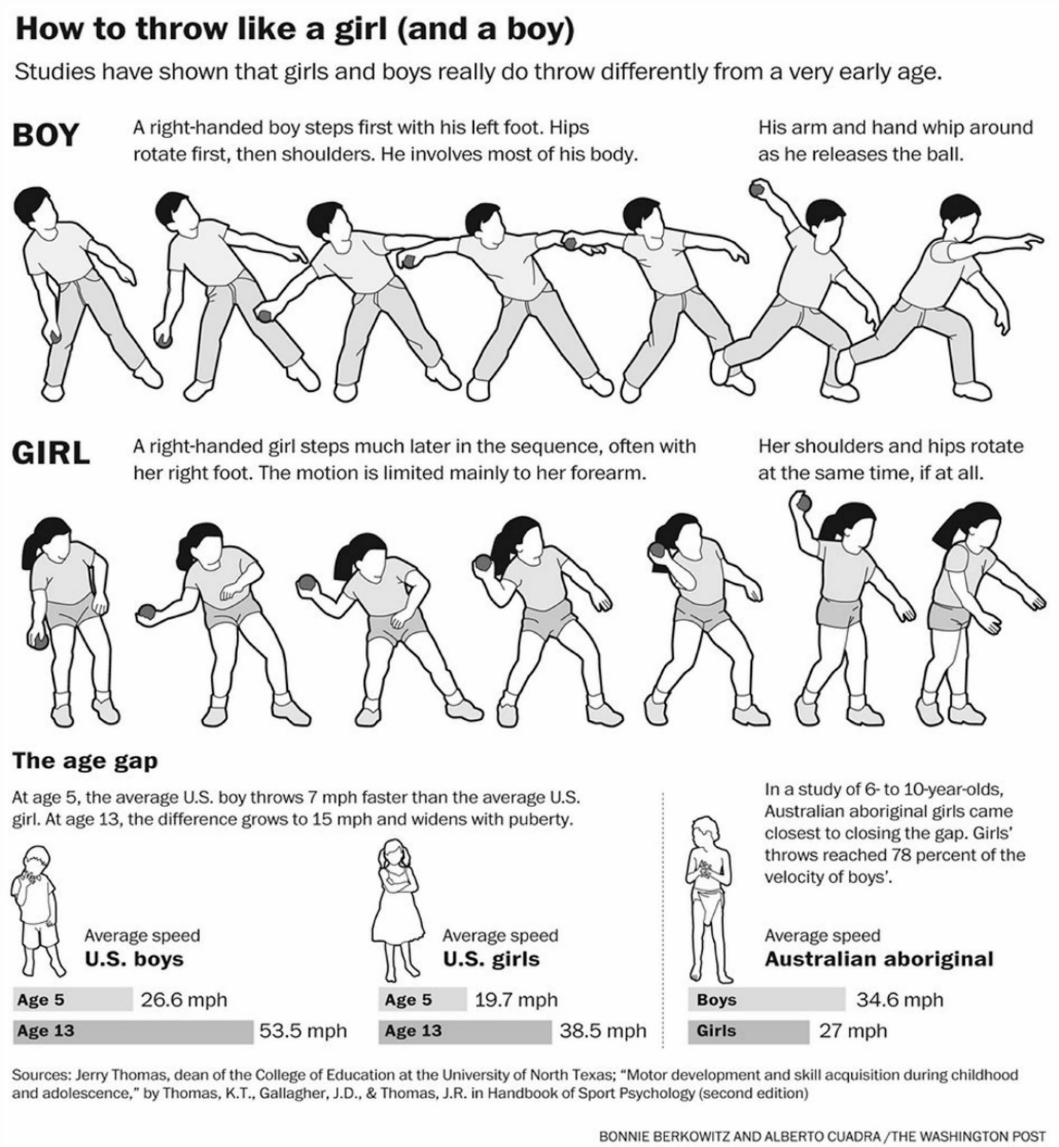
A frequently reproduced “scientific” illustration of the differences between throwing like a boy and throwing like a girl [13]

Since 1980 feminist philosophers–Young included–have avidly discussed and revised her article [6–10]. Early on in the formation of the internet information highway, the phrase established itself as a sexist and homophobic meme. Then a teenage girl named Mo’ne Davis became a celebrity for her 70 MPH pitch [11], and internet images began to change. Today a “throwing like a girl” image search mostly produces images with feminist messaging and videos [12]. Nevertheless, the idea that boys throw further and faster than girls because forceful overhand throwing is an inborn trait has held on with tenacity (see Fig. 1). Two types of arguments—one developmental, the other evolutionary—continue to appear in the scientific literature.

To place gender/sex differences in throwing in an evolutionary context a few evolutionary psychologists posited that present-day differences in throwing distance and accuracy are so great, and (in their reading of the literature) so widespread that the differences must have an evolutionary origin. They predicted that more males than females should have “innate anatomical and behavioral traits associated with throwing.” [14] (p. 91). Similar ideas may be found in [15, 16]. Since my goal in this paper is to address theories of development, I will only briefly consider these evolutionary approaches, which are based on two main assumptions. The first is that the “Man the Hunter” account of human evolution accurately reflects the current consensus among anthropologists and archeologists about the nature of prehistoric human societies and their evolution to the present day. The second is that gender/sex differences in throwing form, distance and accuracy are human universals.

### Man the hunter?

The idea that male hunting shaped human evolution dates, academically, to a 1966 symposium and subsequent book entitled *Man the Hunter* [17]. This idea framed decades of research. But it also received considerable academic pushback. From the beginning, many anthropologists did not buy the argument (see for example [18, 19]). Since 1968 data have accumulated that women in ancient societies often hunted, and that the division of labor was far from binary [20–22]. These more recent assessments demonstrate that the idea that women just gathered roots and grubs, and thus were not selected for throwing prowess, needs to be returned to the drawing board. Of course, it is still possible that Holocene women who hunted used a sidearm throw or trapped their prey, but that argument currently is unsupported by actual anthropological data.

### Is throwing like a girl universal?

Young cited several studies that showed differences in throwing parameters between boys and girls, referring to “The emergence of a standardized overarm throwing motion” as ubiquitous “wherever it has been studied” [23]. (p. 20) He further suggested that this developmental ubiquity provided one justification for speculating about the evolutionary origins of what he believed to be a biologically-based difference. Referring to overhand throwing as a “human universal” (p. 98), Lombardo and Deaner developed a detailed hypothesis to explain the evolution of sex differences in throwing as a male adaptation. They listed the same set of references used by Young to demonstrate the widespread nature of the differences. They also incorrectly cited a 2011 article as claiming “that these forceful propulsive actions are behavioral expressions of masculinity and that masculine children (i.e., mostly boys) may derive greater pleasure from them than do non-masculine children (i.e., mostly girls)”. (p. 98) In fact only one of the three authors cited made that claim and they did so in a 1997 article on the “masculinity of propulsion”.

Given that the above authors cite roughly 10 research publications in support of the claim that sex differences in throwing form and velocity are ubiquitous and possibly universal, I decided to tabulate some of the basic features of each of these papers. Did each measure the same phenotype? Were the ages of the children comparable from one country to the next. Did they always compare boys and girls? The results of this effort are offered in Table 1.

**Table 1:** Features of studies cited in claims that sex differences in throwing are ubiquitous, perhaps even universal

Country	Sample size	Sample Age	Method	Finding	References
Senegal	348 (168 boys, 180 girls)	5–13 years, subdivided by gender/sex, age group and 3 nutrition levels	Tennis ball or softball throw distance	Boys threw further than girls; difference increased with age	[24] (1996)
Senegal	139 (73 boys, 66 girls)	4–6 years, subdivided by degrees, history, and length of malnutrition	Tennis ball throw	Boys threw further than girls; difference increased with age	[25] (1999)
Brazil	71 (34 boys, 37 girls)	4–10 years	Overarm foam ball throw; videotaped. Roberton-style analysis	Boys threw better at all ages; similar to Roberton findings	[26] (2002)
Tasmania	574 (boys, 255; girls 319)	7 and 10 years.	Measured gross motor skills combined; no specific data on throwing	Boys have better gross motor skills at ages 7 and 10	[27] (1997)
Japan	180 (boys 81, girls 99)	3–9 years	Tennis ball, filmed and analyzed throwing form, speed and distance; used Wild’s stage categories	Age 3–7 no statistical sex diff. in throwing speed or distance; attributes diff. after age 7 to culture of father-son ball playing	[28] (1983)
Nigeria	341	3–5 years	Administered a battery of motor tests	No standard deviations in youngest children; in older ones boys somewhat better than girls	[29] (1986)
Mexico	120-178 boys; 89-187 girls	6-15 years	Softball throw for distance	Not concerned with sex differences although separate data by sex.	[30] (1987)
Papua New Guinea	30-47 boys; 28-37 girls	6-16 years	Softball throw for distance	Not concerned with sex differences although separate data by sex.	[30] (1987)
Germany	52 (28 boys, 24 girls)	13–14 years	Tennis ball throw, filmed and velocity measured	Sex diffs between German girls/boys but looks as if national diffs, not analyzed (German boys throw like girls?)	[31] (2004)
Aboriginal Australia	30 (15 each)	10 in each age group (6, 8, 10 year old)	Tennis ball throw, filmed and velocity measured	“The differences in throwing mechanics and ball velocities were less in Aboriginal Australian boys and girls than that observed in other groups of children.”	[32] (2010)

Table 1 highlights the fragility of claims that sex differences in throwing are universal or even ubiquitous. One-off studies, scattered over a thirty-year period and performed in 8 countries do show differences. The studies cited for Mexico and Papua New Guinea separate aggregate data for the boys and girls but do not comment or offer statistical information on sex differences, as the authors were interested in overall cross-cultural differences. Given that the studies used a variety of assessment methods and the age range of the children varied widely, it seems unwise to draw worldwide conclusions. Nor does it seem possible to use this data set to infer much about the underlying factors which generate observed sex-related differences.

Using large sampling methods, ethnographers can and do make claims about universal traits. One classic example, in which the authors attempted to quantify the idea that all societies have an organized division of labor by sex, started with an ethnographic database of 186 cultures. They coded over 9000 activities and from that large data set drew their conclusions [33]. They found universal patterns or trends, but also a fair amount of variation, a point elaborated upon further by Rosaldo [18]. In the case at hand, the claim that sex differences in throwing are ubiquitous wants badly for substantive data. In lieu of such data, which also presumes patterns of motor development that exist independently of specific cultural patterns of motor training. Why not instead ask some very basic questions about bodies and ball play? With this, I turn from evolutionary to developmental theories.

### Developmental theories

#### “Throwing is different” [34] (p.276)

In 1985 Thomas and French published a meta-analysis of reported gender differences in motor performance in children and adolescents [34]. After compiling data from 65 publications and looking at performance on 20 motor tasks, they concluded that gender differences in performance existed on 12 out of 20 of these tasks. Eight of these 12 had small effect sizes, and the degree of difference changed with age in ways that suggested that they were environmentally produced. That left two—throwing distance and throwing speed. For these—and again based on only a single study at the youngest ages–the effect sizes were large at 3–4 years and grew throughout childhood.

Thomas and French calculated a mean effect size of 1.5 for differences in throwing distance for three year olds. Although it is not possible to tell how many studies this conclusion was based on, the pooled N was over 300 for each comparison group. Between ages 4 and 15, the effect size for differences in throwing distance more than doubled. Thomas and French calculated that the mean effect size for differences in throwing velocity remained at 1.5 from ages 4–6 and then increased regularly to 3.5 at age 15.

To draw inferences about the roles of environment and biology in producing gender/sex differences in motor performance in pre-pubertal children, Thomas and French emphasized the shape of the curves in which they plotted mean effect size (Y axis) by age. For tasks such as grip strength or vertical jump, for example, until the onset of puberty effect sizes for gender/sex differences hovered around 0.5. The authors concluded that “any gender differences in performance prior to puberty on these 10 tasks (balance, catching, dash, grip strength, long jump, pursuit rotor tracking, shuttle run, sit-ups, tapping, and vertical jump) are mostly environmentally induced.” ^34^ (p. 275). For throwing, the effect size of 1.5 at age 3 and 4, and the fact that the degree of difference continued to grow during childhood, led them to argue that the difference had to be primarily biological. They were especially struck by differences already measurable at the young age of three, a point I consider when I present my own developmental data.

### How does difference develop?

How do differences in throwing force, speed and form arise? I am particularly interested in studies of infants and toddlers younger than age 3. So much motor development happens from birth (and in the last trimester) to toddlerhood that I find it unimaginable that this period does not set the neuromuscular stage for throwing. If Thomas and French’s singleton report that gender/sex differences already exist in three-year-olds were to be confirmed, then a next step would be to figure out how those differences came into being. They are not there at birth, when infants cannot even lift their head.

Wild (1938) conducted a cross-sectional study of overhand throwing in 32 children. She paired a boy and a girl at 6 month increments—from 6 months to seven years, and at yearly intervals from 7 to 12 years [35]. She filmed each child in “a carefully arranged throwing field” (p. 21) with distance markers and an electric timer. Each subject attempted three overhand throws. Wild collected distance and throwing form data from the filmed trials. She characterized her results by developmental progress—from stages 1 to 4, designating stage 4 (what today would be called throwing like a boy) as the mature form achieved by all boys by age 6.5. At the same age the girls showed what Wild thought of as a developmental lag, remaining in stage three or earlier.

Over a period of more than 30 years, Roberton and colleagues conducted longitudinal studies of the development of overarm throwing. Although they studied older children, I pay some attention to these investigations because they articulated and tested specific developmental theories. Wild and others suggested that all of the body components involved in throwing passed through specific developmental stages as a single unit. Roberton separated throwing into aspects that, potentially, could develop at different rates. The three major components—the humerus action, the spine and pelvis action, and the sidearm channel–each had different sub-components [36, 37]. Roberton filmed 10 throwing trials per child sequentially in kindergarten, then first, then second grade [37].

While Wild reported that boys had mature throwing form at six and a half years, Roberton wrote that even at the end of the second grade (age 7) “the majority of children…were still showing primitive to intermediate level movements” [37] (p. 174). She also found that the motor components of throwing developed at different rates, concluding that “the issue of ‘stages’” should “be confined to the ordering within the components rather than to the total body configuration” (p. 174).

To delve more deeply into the question of sex differences, Roberton and colleagues conducted a longitudinal study of overarm throwing velocities in boys and girls aged 5–7 [38]. Even in kindergarten (age 5) the boys threw a tennis ball significantly faster than the girls, and yearly testing showed that by second grade (age 7) the difference had increased by 1.5 times. By age seven, for all three of the tested movement components, the girls averaged one or more developmental stages younger than the boys. One speculation for the origin of the difference was that girls had fewer and different types of throwing practice.

Roberton and colleagues refilmed these same children when they were in the 7th grade (age 12) and assessed developmental changes in throwing movements involving the humerus, forearm and trunk. She and co-authors also described and quantitated the throwing experiences reported by both the girls and the boys [39]. Between ages 7 and 12 the throwing velocity for the boys showed an average increase of 5.04 feet/sec/year, compared to the girls’ average increase of 2.94 feet/sec/year. In terms of developmental years, their results meant that the boys were five years ahead in ball velocity throwing. There was, however, group overlap, such that the top girl and top boy threw at nearly equivalent speeds (87 feet/sec vs. 92 feet/sec).

Until 1982 the literature on motor development had suggested that children aged 5–6 had fully developed gross motor patterns. Then Halvorson et al. (1982) looked at the development levels for humerus, trunk and forearm components of throwing. By age 12 none of the girls, but 31% of the boys had advanced developmental form for the trunk action component; 12% of the girls but 41% of the boys had reached advanced forearm form, and 20% of the girls, but 82% of the boys had reached the advanced humeral action step. Roberton and colleagues emphasized three conclusions: throwing components developed at different rates for boys and girls, but they followed the same developmental sequence. Boys advanced more quickly, and, contrary to earlier reports, the 13-year-old boys were “far from having ‘mastered’ or ‘developed proficiency’ in overarm throwing”. (p. 203)

Halvorson et al. (1982) interviewed each child individually to assess whether and for how long they had participated in organized ball games such as the Little League. Most of the boys (20/22) compared to fewer than half of the girls (8/17) participated for 2–5 years in some form of league ball play. There were similar differences in overarm throwing practice during spontaneous play during “the outdoor season”. Differences in levels of practice, then, were significant and included both organized sports and daily play activities. (It is hard not to be a bit chagrined that I first pointed out in 1985 that play and experience differences might affect cognitive skills such as spatial perception, and spatial perception is often linked to throwing accuracy. But the possible connection with Halvorson et al.’s finding of experience differences has never been made [40] (see esp. p. 36).

Thomas and French’s highly-cited meta-analysis of gender (sic) differences in motor performance set up the analytic or causal problem in terms of the relative contributions of nature and nurture (biology and culture). With only a few exceptions [8, 41–43], that has been how scholars have framed the issue. In 2001, however, Roberton and Konzak significantly restructured the question. Again they video recorded children at ages 6, 7, 8 and 13 years. Subsequently, they coded the films for developmental action sequences “of the humerus, forearm, trunk, stepping stride and length” [43]. (Abstract, p. 91). Using a multiple regression analysis, they assessed how each of the well-defined movement qualities, and the undefined idea of gender^1^ contributed to variance in the development of ball velocity in their total sample. If they entered gender (their term) as a predictor of throwing velocity last in their regression analysis, they found that it was “redundant with developmental level” (p. 101). In other words, 98% of the variance in throwing speed was accounted for by variance in humerus, trunk and forearm, and stepping and stride performance. The relative contributions of these motor components changed with age, but gender as a stand-alone component never accounted for more than 2% of the variance.

Roberton and Konczak wrote “While the search for an explanation of gender differences in throwing needs to continue, the present study narrows that search to the question of why throwing patterns and, therefore, ball velocities are delayed in some girls up through the age of 13 years” (p. 101). Their finding emphasizes that using “gender” as a measurement factor often obscures the events and behaviors that the word stands in for. In recent years methods scholars have emphasized this point by dissecting the events that “gender” stands in for [44, 45].

Dissecting the relevant factors that make up “gender” as a variable is an important step. But it still does not account for an organism’s long-term developmental history. In the following sections I push for a further reframing. As an example, I offer a preliminary analysis of ball play from 3 to 15 months. Rather than using specific tests of velocity or throwing form analysis, I observed ball play “in the wild”, that is, during day-to-day play bouts, both solo and in interaction with the infant’s mother.

## Methods and results

Motor skills develop out of the daily repetitive activities of infancy and toddlerhood (see, for example [46–49]). With older children, targeted training may supplement or advance already-developed body habits. In the exploration described in this article I bring to bear an extensive and vibrant literature that combines a “natural history” approach with sophisticated technology (body cams, room sensors, heart and vagal nerve monitors etc.) to study infant development as a complex developing system [50–60].

My study is exploratory and primarily qualitative. Detailed investigations with small samples are, I should note, a time-honored approach in developmental psychology [61–64]. I started with ten videos of children aged 14.5–15 months (five labeled as boys at birth and five as girls). The videos, originally recorded by a colleague studying the development of temperament traits in infants, run for 30–45 min. A videographer followed each child as they played with toys—sometimes alone and sometimes with their mother--explored, ate a meal, got washed, diapered and dressed. Using NVIVO (https://lumivero.com) a software package designed to analyze qualitative observations, I coded their basic motor abilities, what interested them as they moved around, how the mother interacted with them, and what interested her (on their behalf). Details of the larger study sample from which these 10 infants were taken have been published elsewhere [65, 66].

At 15 months, seven out of 10 of the infants were walking; two were crawling and one was crab-walking; they were all finger feeding and holding cups and bottles for themselves. Regardless of mode of locomotion, they moved around independently and were relentlessly curious. Comparing these 10 babies, all so close in age, reminded me how individually quirky children are. One of the boys was quiet and contemplative; one girl had advanced motor skills, while one of the boys took up the rear in terms of motor skills. One of the girls had a defiance thing going months in advance of the classical terrible twos, but several of the kids readily complied with whatever their mother asked.

The infants had dozens of play object choices, from the pots and pans in the kitchen cabinets, and magazines on the coffee table, to all of their own toys, to a set of toys the videographer brought to each home visit. At times the mother, on the request of the observer, stayed in the background while the child explored and played on their own. At other times the mother chose a toy or activity and tried to engage the child in a joint adventure. And at yet other moments the mother used a toy to distract the baby as she bathed, diapered, clothed, or fed the child. Kind of like daily life.

**Fig. 2:**
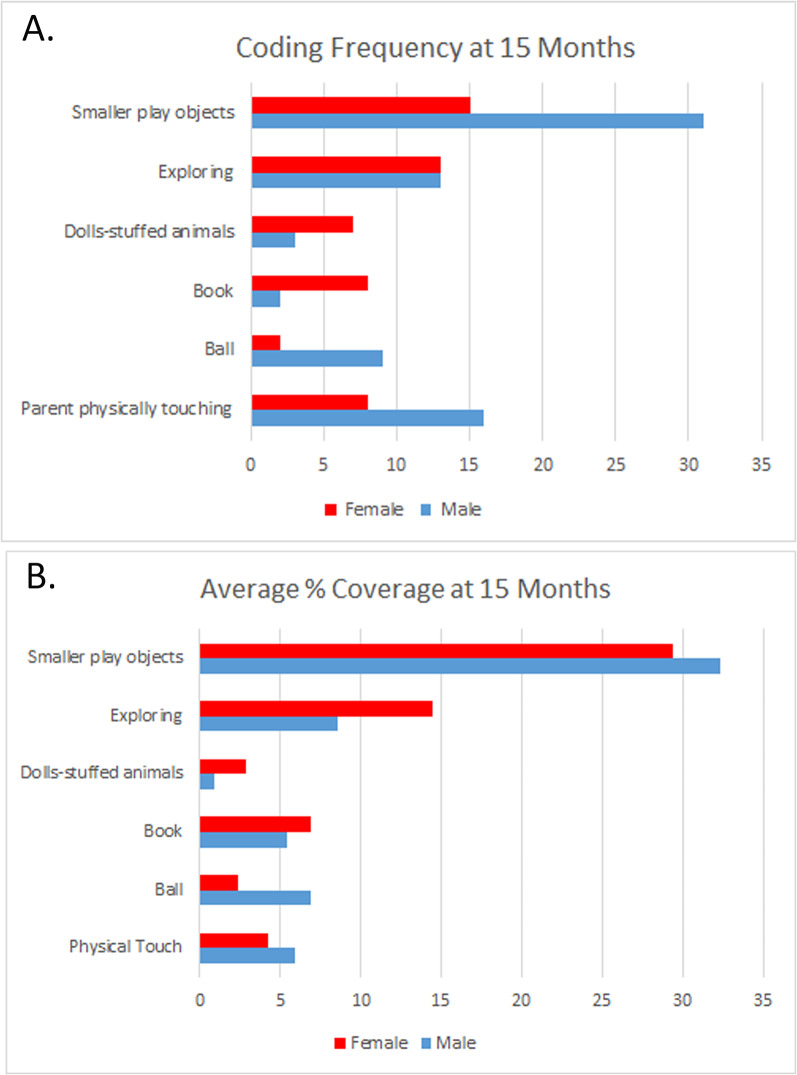
Play interactions in ten 15-month-olds. (**A**) Coding frequency-the number of times I observed an event. (**B**) The average percent of total observation time

At 15 months, for these 10 babies, I found certain gender/sex-related differences in behavior patterns. Figure 2 shows some of the behaviors I coded, second by second, as I reviewed each tape. The top graph (Fig. 2A) illustrates how often I coded a behavior during the course of a recording session. None of the sessions were exactly the same length, however. Therefore, in Fig. 2B, I used NVIVO to calculate the average percentage of time (out of the total video length) spent by girls, boys and their mothers in physical touch within the dyad, and in interaction with balls, books, dolls, stuffies, smaller play objects, and exploration through movement from one place to another.

*Patterns of exploration*. I started conventionally, by averaging instances of behavior and comparing group differences. The boys and girls engaged in the same number of exploration bouts (2 A), but the girls—all of whom were walking, compared to only two of the boys—averaged 1.7 times longer in any particular exploratory bout. Because on average, the girls explored for longer periods of time, they stopped to play less frequently, and when they did stop, they averaged slightly shorter play periods before tooling off to their next activity. The babies spent more of their time exploring the properties of small objects—shape, noise making abilities, hardness, taste, etc., than they did on items that have come to be seen as an aspect of gender/sex [67].

As previously reported [65], on average, boys and their mothers touched one another twice as frequently, and the sessions averaged almost one and a half times longer than for girls and their mothers. I reviewed each of these coding events individually, and although some of them involved rough housing and tickling, most were sessions of affection, the latter often initiated by the baby.

Since this article focuses on the phenomenon of “throwing like a girl” I next looked more deeply into ball play. Thomas and French wrote emphatically that “throwing is different” (p. 276), even as young as three years of age. More recently, Todd, Barry, & Thommessen reported that boys as young as nine months played more on average with male typed toys such as balls, diggers, and trucks, compared to girls, who preferred female-typed toys such as dolls, cooking pots and teddy bears. They observed these differences in a nursery setting with a specified set of toys and without parents in the mix [68].

Returning to Fig. 2B, I note that mother-son dyads played with a ball for 2.5 times longer than did mother-daughter dyads. Given the small sample, I saw this not as a statistical claim, but as an invitation to look more qualitatively. At this point I made a methodological switch to detail descriptions of individual behaviors. When the first ball vignette began for 15-month old Stephen^2^, he was walking around looking for something, and his mother said “Oh here’s your ball” as she handed it to him. Then they had the following 6.7-second exchange:

Mom: “Dunk it!”

Stephen: “Wha?”

Mom: “Dunk it!”

Stephen” “Wha? Wha?”

Mom: “Yeah. Dunk it!”

Stephen: “Oomph” (as he dropped the toddler-proportioned basketball into an open basket on the floor before walking off without retrieving it.)

Mother (to his retreating body): “Are you playing basketball?”.

The mother of another boy, Jimmy, aged 15.25 months, went to some effort to engage him in a ball game. At the start of a 3.3-minute interaction, Jimmy crawled away from her, with a soft, nubby ball clenched in his mouth. “Come here you silly kid. Jimmy!” she said, and he turned back to her. She got down on her hands and knees, took the ball in her mouth, and in a move worthy of an excited puppy begging to play, shook it at him to get his attention before dropping it to the floor. The ball rolled towards him, he picked it up and threw it to her. “Yaaay” she exclaimed as she rolled it back and he threw it again. “I got it! Ready?” Jimmy let out an excited shriek. “Yaay. Ready? Huh? Ready?” By this time Jimmy had crawled off a little distance and sat facing her expectantly. Apparently, they had played this game before. One more roll and throw before he got distracted and crawled off. The mother deployed several tactics to re-engage him in the ball game, finally grabbing and tickling him, before bouncing the ball off his head several times, saying “Bonk! Bonk! Bonk!” each time. But he was done with the ball and turned away.

In contrast, at 14.6 months Susie found a small rubber ball on the floor, held it out to her mother and then bounced it to her. “Oh thank you” said Mom as she bounced it back to Susie. The little girl ran after it, retrieved it from under some furniture, and as it rolled past a bag of toys, got distracted. End of the 30 s ball game. As a final comparison I watched Jane’s mother engage her over a 1.4-minute stretch by setting up a four-way interaction between mother, child, the ball and their puppy whom I’ll call Rover.

“Wanna throw the ball for Rover?” Mom asked as she handed Rover’s ball to Jane. And then “Good. Yaaay!” as Jane threw it, while afterward complaining vocally because Rover wouldn’t give it back to her.

"That’s OK, honey. That’s his ball. There it is. Throw it again".

This sequence repeated about three times and then the mother threw the ball to Rover while Jane got distracted by another toy.

What might we think about these episodes? For both boys the mother facilitated the play by handing her son a ball or really working it to get him play a two-way game of throw and roll. Stephen’s mother framed one interaction as a game of basketball. Jimmy’s mother got down on all fours and clenched the ball in her mouth to get him to play ball with her. In contrast, even though Susie’s mother responded politely when Susie bounced the ball to her, the mother made no further effort to keep her engaged with this toy. For Jane, the ball play involved throwing, but as part of an interaction with the dog (who owned the ball) rather than a game of catch with the mother.

To figure out how these different play patterns arose, I next looked at a fuller age sequence of ball-playing experiences. I watch weekly tapes for Stephen (37 tapes ranging from 2.5 to 15 months) and Susie (30 tapes ranging from 3.5 to 14.75 months) to find out when I could first observe them with a ball. I started at 6–7 months before the first ball interactions appeared, and coded the length and number of events of ball-playing (either alone or with the mother). I binned the data into 2-month groups. I use frame grabs from some of the videos to illustrate certain observations. To create the figures I altered each frame using an online program called Vector/Dad Photo to Sketch (vectordad.com) to minimize the identifiability of clothing colors and facial details. To further anonymize the frame grabs, I used Photoshop to remove irrelevant items such as other toys, furniture, and even room windows and to otherwise transform the setting.

At 15 months Susie and Stephen had similar motor skills, and in terms of personality seemed pretty middle of the road—neither super-quiet nor off-the-charts excitable. I wanted to know how often in the past they played with balls, how the play began, how long a game might last, and what specific skills were practiced in a typical encounter. Doing a deep dive into just two children does not, of course, reveal some general law of gender/sex and ball playing. But it can show how these two individuals—Stephen and Susie–developed stronger or weaker relationships to ball-playing, how they adapted their bodies to ball-play, and what the social context of ball play might be for them.

**Fig. 3:**
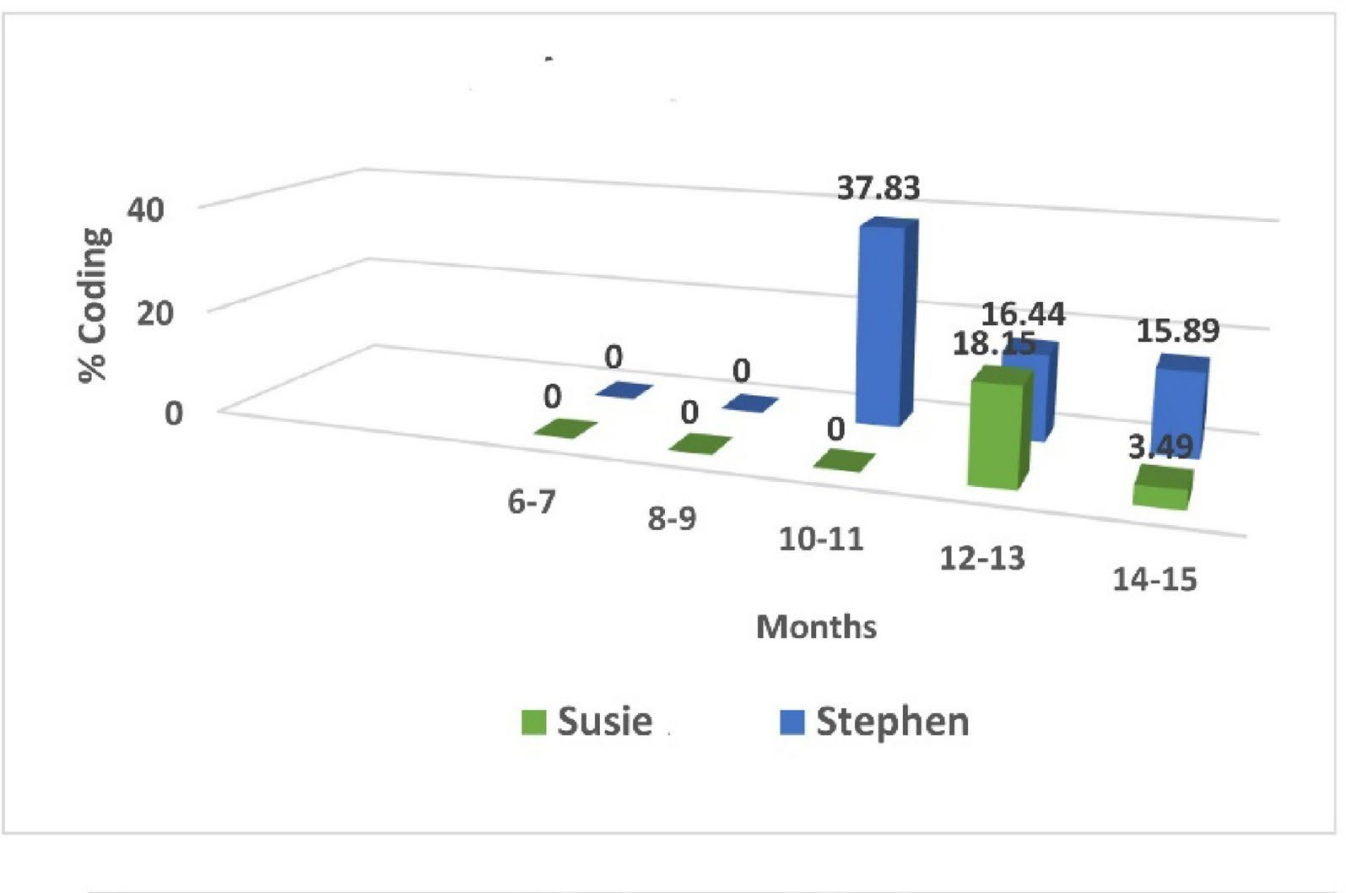
Susie and Stephen learn to play ball. Y axis = the percent of time (of all coded play items) spent playing ball. The X axis = age in months

**Fig. 4:**
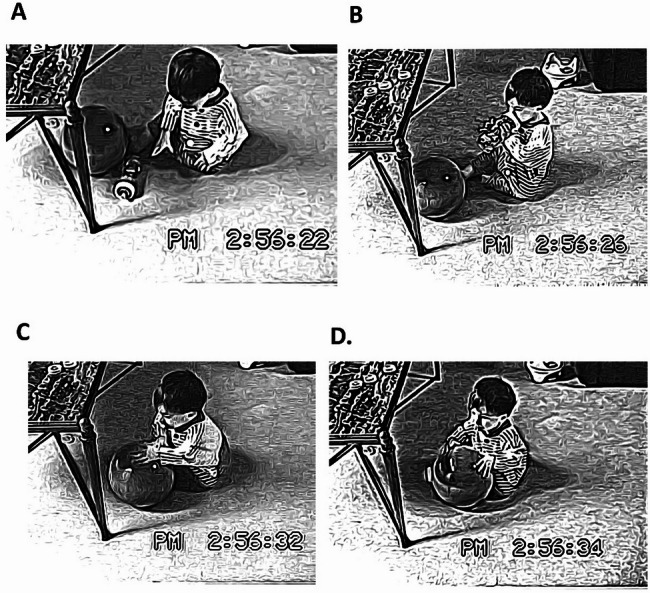
Stephen meets the ball at 10.7 months. See text for description of each panel

For Stephen an overview of his ball playing (see Fig. 3) brought to mind the ironic phrase “vote early and often”. Starting in the 10th month he and his mother played ball regularly and sometimes for bouts lasting as long as four minutes. When, at 10.7 months, ball play first showed up in his tape set, the episode seemed almost accidental. As the scene opened he was standing in front of a table, his elbows propped on it in a way which both supported his standing position and allowed him to grasp a bottle from which he drank. A blue kickball the size of his torso could be seen lying under the table out of his line of sight (Fig. 4A). As he sank to the floor, however, he retrieved his dropped bottle, noticing the ball but returning to suck on the bottle. Twenty-five seconds later he touched the ball that sat directly in front of him, but continued to sit with the bottle (Fig. 4B). Finally, 40 s after seeing the ball (and having dropped the bottle) he reached for it, pulled it to him (Fig. 4C) and patted it on the top first with one hand then the other (Fig. 4D). The ball rolled off a little but he reached and retrieved it. Then, moving the ball, but with little control over it, he accidentally sent it rolling to the side where it stopped a short distance away. He did not crawl after it, but got interested in a different nearby item, and I ended the code. Total time spent: about one minute.

**Fig. 5:**
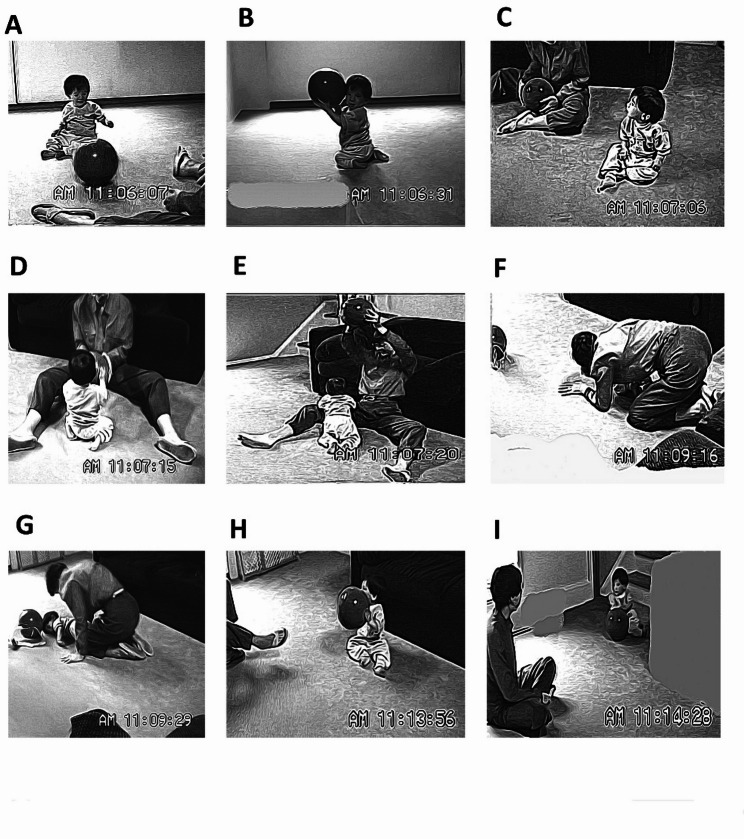
Stephen and his mother play ball at 11 months. Timing, narration and panel references are given in Table 2. Actual time stamp when recorded is visible in each image

Half a month later Stephen’s ball skills had become more complex. He crawled to the ball, kneeled, picked it up, and from a kneeling position threw it with both hands. Thus, he learned the two-handed pick-up and throw even before he could walk. He also had somewhat better directional control when he rolled the ball. In addition to playing alone with the ball, it became part of a game with his mother, something she encouraged. In the several-minute episode, Stephen’s mother riled him up with physical play as a ploy to keep him engaged with the ball. I offer color commentary and a transcript of this sports event in Table 2. There is a six minute play stretch, a maternal sneezing fit and then a reintroduction of the ball. Throughout, the mother initiated the ball play, and Stephen responded with brief moments of interest (Fig. 5).

**Table 2:** Transcript and timing of play period between Stephen and his mother with references to images in Fig. 5

Timespan (minutes from start of tape)	Content
7:39.3–8:22.9	Before the segment with the ball he keeps crawling to a trash can. She says no twice and moves him away, but he returns. So she says “ok I guess we have to go into another room don’t we?” Cut to the living room and she is standing and letting the dog out into the yard while holding the ball. He is sitting on the floor gazing up at her.
8:20.2–8:29.4	She walks a little distance from him, sits on the floor with her legs spread facing him and the ball between her legs. “OK, come on”. He vocalizes.
8:22.9–8:30.3	She rolls the ball in his direction (Fig. 5A) and it rolls past him and he follows it with his body (top half). “Where’s the ball! Huh (inhale). Go get it!”
8:34.7–8:45.8	“Go get your ball. Get the ball.” He looks at her. “You want Mommy to go get it? You don’t feel like chasing it?” OK. And she reaches out and retrieves it to between her legs.
8:45.8–8:49.9	“Ready?” She rolls it past him, but this time he reaches for it and starts to crawl after it.
8:49.9–9:02.4	He grabs it, pats it, and picks it up with both hands. (Fig. 5B) He throws it, but not toward her, as he happens to be facing away from her. She says “throw it to Mommy. Uh Oh, Good throw, but wrong direction.”
8:58.1–9:04.6	Stephen incidentally pushes the ball towards her and she reaches for it and says “here you go” as he crawls past her.
9:04.6–9:16.2	He looks up at the videographer; the dog barks, and he sits on the floor near his mother but not in “catch” position. She is still holding the ball between her legs. “Is that the dog?” (Fig. 5C)
9:16.2–9:30.5	He sits kind of staring into space and finally she draws his attention by saying “Yoo-hoooo”.
9:30.5–9:35.4	He looks at her. She says “Oh hi” and he crawls over and hugs the ball which is still between her legs. (Fig. 5D)
9:35.4–9:43.8	Now facing each other she also puts her hands on the ball and shakes the ball while he is holding on. He pats it a couple of times and she playfully says “I got the ball. I have it. You can’t have it. I want it”:(while shaking it loose and raising it up over her head. (Fig. 5E)
9:44.2–10:06.6	He then looks at the out-of-reach ball and crawls away. “Where you going? You’re not gonna play with this?” She lowers the ball. The dog barks at the patio door again and she says “Where’s [the dog]?” as baby looks in dog’s direction to see [the dog].
10:06.6–10:19.6	He turns around and crawls back to Mom and takes the ball in both hands and holds it up and then throws it. “Good throw”. as mother retrieves the ball. (Fig. 5F)
10:19.6–10:26.5	Mothers moves out to face him. says “ready?” and then rolls the ball to him. He takes it and sits kneeling with his arms resting on top of the ball.
10:26.5–10:32.8	Watches as his mother gets up to let in the dog who has been continuing to bark. As she returns he pats on the ball and it rolls toward her as she sits down and picks it up.
10:28.1–10:40.5	She holds it up in front of him and says his name. and then bounces it to him. He gets it again and again pats the top of it.
10:59.1–11:07.0	He pats it until it bounces a bit and rolls towards his mother, possibly with some intention but not a lot of motor control.
11:02.1–11:17.3	Wanna give the ball to [says his name]? Then she rolls-bounces it to him. It kind of bounces off him and back towards the mother and he crawls after it and vocalizes, touches it and it kind of rolls away. He does not chase it but crawls onto his mother’s leg while looking at the camera.
11:17.3–11:25.6	Mother initiates excited play. “Where you going? You’re not going over there. I’m gonna get you” while she grabs him and puts him on the floor in front of her and gets up on her haunches. The ball is in view a few feet in front of him. he is on his belly on the floor (Fig. 5G->5
11:25.6–11:46.4	She completely covers him with her body, nuzzling him; but he also seems to try to push her away. Then she starts pushing him with her body towards the ball, scooting him across the rug. “Get the ball. (a noise from the baby?) Move,” as she scoots him to touching the ball. She makes a kind of growling sound as she pushes his head into the ball, turns him over (the balls rolls away a little) and she nuzzles her head on his belly. He is laughing but also maybe crying. An ambivalent sound. (Fig. 5I)
11:46.4–12:23.6	More nuzzling and physical play rolling around on the floor with him making strange sounds. Ball in view. Then she stops, strokes his face and wipes his mouth. “Whattya got? Dog hairs? and lipstick all over your face? Boo!
12:23.7–13:40.3	More physical play with him on his back and he is giggling now. He grabs her hair. “Gimmee my hair. I want my hair” More giggling and physical play. Plays with feet. Claps his feet. Boo!. Wanna ride a bycicle?. Does bicycle legs.
13:40.4–13:42.2	Then she sneezes, he sits up and moves away and the dog returns to the picture.
13:42.2–14:47.0	Play with the dog. He laughs. She gets up to deal with her sneezing fit, while he crawls over to the camera and pokes his face right in it. She starts to call after the boy again.
14:47.1–15:16.1	Sound of bouncing ball as she tries to reinterest him. He watches.
15:16.1–15:27.5	Then he starts to crawl to her and she offers it from a standing position. He reaches with one arm. “Want it?” she offers from a standing position then hands it to him. He takes it and throws it as she sits in front of him and receives the throw. “Very good”. (Fig. 5J)
15:27.5–15:50.8	She throws it and it bounces off him but he gets it then crawls past it and towards the stairs? “Where you going?” the mother asks? He sits next to the ball and she now tries to gain his attention with a different toy.(a soft plushie truck).
15:50.9–16:24.6	She squeaks the truck and he pats the top of the ball which he is now sitting next to. They are several feet apart. then he turns away and toward the stairs. she tries again with the truck but he is off climbing the stairs. (Fig. 5K)

**Total time: 8:85.3 Minutes**

**Fig. 6:**
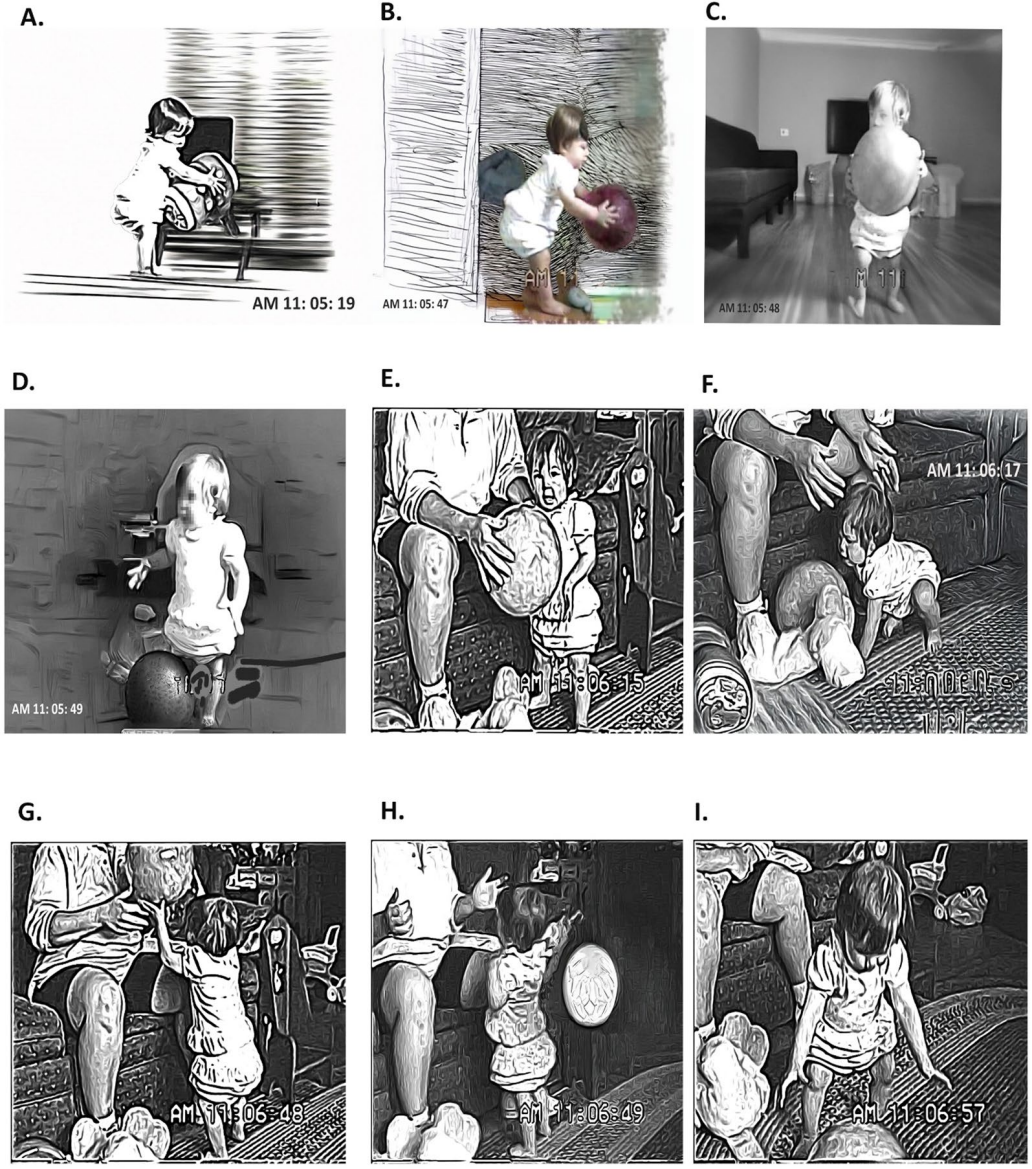
Susie and her mother play ball at 13.6 months. Timing, narration, and panel references are given in Table 3. Time stamp at moment of recording visible in each image

While Stephen alone or with his mother played with the ball frequently and for relatively long periods at 10–11 months, we did not find a ball-playing sequence for Susie until 13.6 months. At the time she was also just learning to walk and was unsteady in the upright posture. The transcript of this first ball episode, which lasted a bit over 3 min, is in Table 3 and the accompanying still shots in Fig. 6.

In this first observed ball episode Susie could barely lift the ball to the middle of her chest before falling down (Fig. 6D-F).

**Table 3:** Transcript, description, and timing of play period between Susie and her mother with references to images in Fig. 7

Timespan in minutes from start of tape	Content
9:30.3–10:22.1	Susie wanders looking for something to do, then picks up a squeaky toy and teeters around using both hands to make it squeak. She brings the toy to her mother who says:
10:22.2–10:33.0	“You want to get your ball. Susie, can you go get your ball? Get your ball, " and baby moves off in the direction of her ball.
10:33.1–10:38.8	She leans over and picks up a crawl roller with both hands (Fig. 6A). She tosses it, kind of stiffly at the elbows as if it were a ball. Off camera the mother says “that’s not your ball. THAT’s your ball.”
10:38.9–10:42.7	Baby picks up the roller again, looks at her mother, walks toward her and tosses it again. Mother says “ok (inaudible).
10:42.7–10:51.8	Susie picks up the roller and drop/tosses it several more times, moving towards her mother who is sitting on the couch. She finally hands it to her mother who says “thank you.”
10:51.9–11:20.2	As her mother holds onto the roller, she says “now go get the real ball” and Susie crosses the room again to pick up a pink kickball (Stephen’s was blue). She starts tossing it. Once. Again. Elbows bent at right angles. She bounces it a third time and the reverb from the effort sends her falling on her butt as the ball rolls away. (Fig. 6B-6D)
11:20.2–11:27.2	“I got it” you can hear the mother whisper. Then off camera you can hear the ball bouncing as the mother dribbles it in place while sitting on the couch. Susie moves towards the ball.
11:27.2–11:40.0	Mother hands her the ball and baby stumbles as she takes it and squats to pick it up again. She has a hard time trying to stand while holding onto the ball (Fig. 6E-F) and the ball rolls away as she stands up.
11:40.0–11:52.3	Once upright she walks over to it, vocalizes twice, picks it up (Fig. 6G) and returns it (with a tiny throw) (Fig. 6E) to her mother who says “thank you”.
11:47.4–11:59.5	The mother starts to toss the ball up in the air and catches it. saying “up in the air! up in the air!” while gazing at Susie who is locking eyes with her. Mother tosses the ball in the air three times.
11:59.5–12:05.1	As Susie reaches for the ball mom says “Want it?” and one has the impression that Susie wants to try the air tossing trick. But she can’t manage it and ends up tossing the ball backwards over her head. (Figs. 6G-H)
12:05.2–12:23.8	Mother says “Oh. You let it go up in the air”. Baby walks to retrieve it (Fig. 6L) and picks it up and walks back to the couch, where she stumbles, drops the ball and leans down to pick it up. Susie has been looking suspiciously at something off camera (a pet?) and the mother says “what’s the matter. You afraid?? gonna steal your ball?”
12:23.9–12:51.1	As Susie picks up the ball, the mother distracts her to a different activity (although Susie was still on a roll, as it were). “Where’s the balloon?” Mother takes the ball and the reverb causes Susie again to plop on her butt. Mother repeats “Where’s the balloon on your chair?” Susie looks in that direction. Then she moves off and gets distracted by another toy.

**Fig. 7:**
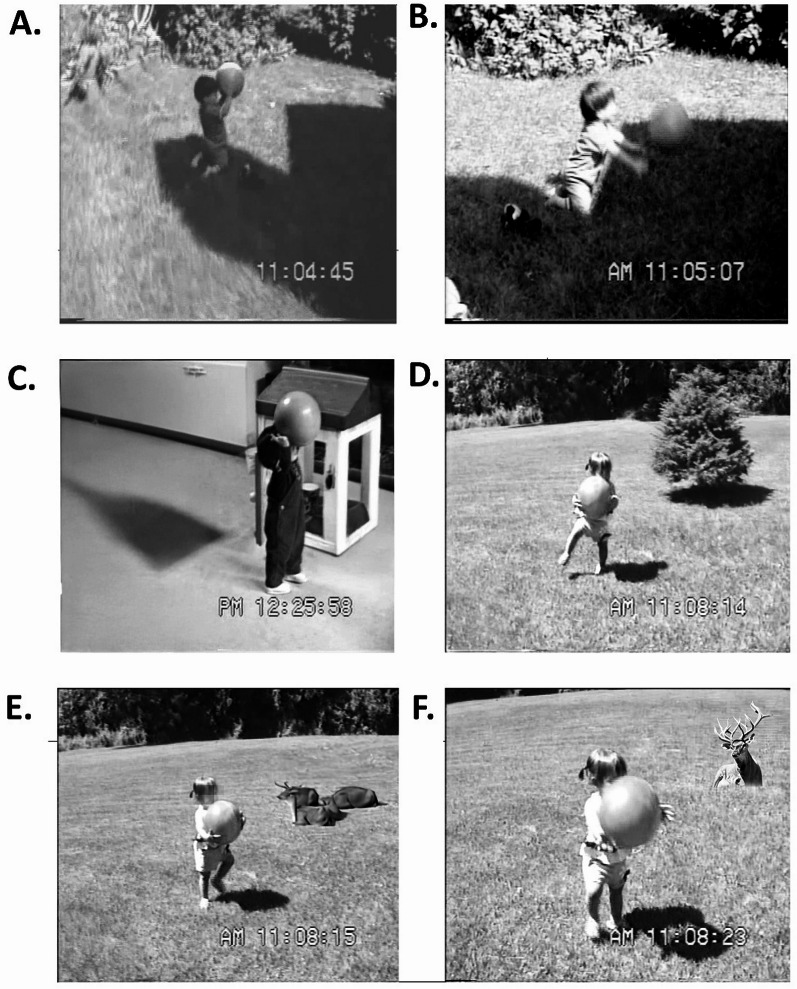
Stephen (**A-B** 13.4 months; **C** 14 months) Susie (**D-F** 13.75 months)

A week later, in the episode illustrated in Fig. 7 Susie had a few successful throws before falling [3]. She could stabilize herself (Fig. 7D) and lift the ball to mid chest, but still threw awkwardly (Fig. 7E-F). Stephen, who in Fig. 7A-B was 13.4 months, stabilized himself in a kneeling position, raised the ball above his head and threw with a strong forward push. In Fig. 7 C Stephen was 14 months, standing, and could lift the ball well above his head and throw it with a strong forward thrust (at which he teetered for a moment and then fell onto his knees and finally sat). For both Stephen and Susie grasping a ball with both hands, lifting it up and throwing it forward is embodied—that is, their throwing techniques are shaped both by their body’s current status (crawling or walking) and past history [69].

**Fig. 8:**
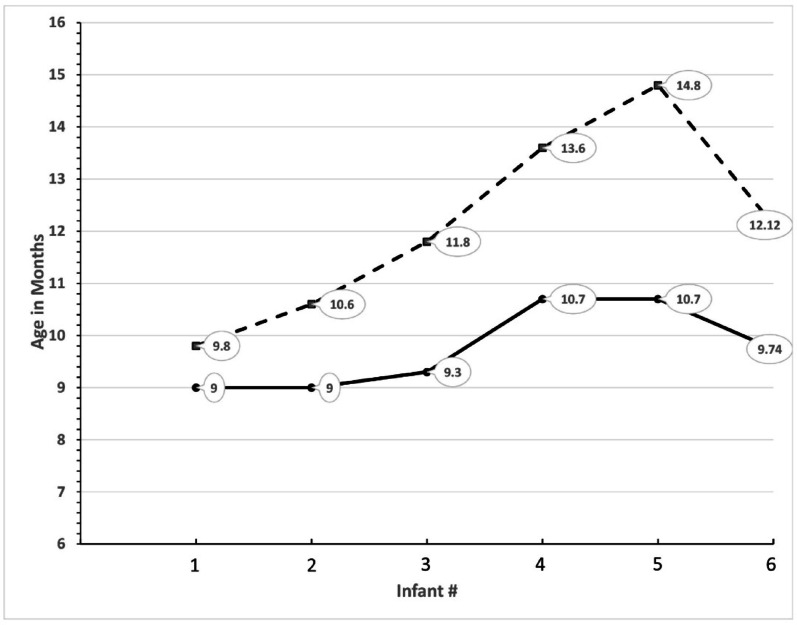
Age at which first ball play observed. Dashed line = girls. Solid line = boys. Balloons are the actual age value. Last data point (#6) = group average

It is reasonable to hypothesize that Stephen could raise the ball above his head, even while standing and a bit unstable, because before he could walk, he had mastered the throwing skill from a more stable sitting and then kneeling position. Susie, by comparison, found it hard to stand and throw the ball at the same time. Perhaps, then, one aspect of learning to throw like a boy or a girl is the age and level of motor development at which one first begins to play ball. To explore this hypothesis, I returned to the 10 children introduced earlier, noting when I first saw them engaged with a ball. The results were striking. As can be seen in Fig. 8, the boys began to play ball as early as 9 months and all had been introduced to the activity by 10.7 months. In contrast the girls were older with three of them a year or more before their first video-recorded encounter with the ball. Like Stephen, none of the boys were walking at first ball, meaning that they became accustomed to handling the ball from a relatively stable seated or kneeling position. Only one of the girls, at 9.8 months was primarily an active crawler. One was a more skilled walker than Susie, and the other two were transitioning to walking at the time when I first observed them being introduced to the ball. The only girl who was still crawling when first seen playing ball in a sitting position with the mother did not, by the time she was a skilled walker, play with the ball even though it was in plain sight in the playroom. Nor did her mother try to initiate ball play. Instead, the mother encouraged her daughter to put items (a doll, a plastic soda bottle) into a toy shopping cart and push it around. Several times the mother pushed the ball aside because it was in the way of the other game.

## Discussion

“Throwing like a girl” is one of those hubs, nodes, cultural knots, or entanglements that does way more work than is justified by robust theory or science [70–72]. Aimed at men and boys it can serve as a homophobic slur. Directed towards girls and women it becomes a take-down of their competency. In the academic and sports world the phenomenon of "throwing like a girl" has long been supposed to support the nature side of a nature or nurture paradigm in which many sex-related behavioral differences are seen as both innate and disabling [see 14]. Sports and physiology researchers continue to pursue the causes and descriptors of throwing in boys and girls [73, 74]. Feminist theorists have mounted a set of robust counter-arguments aimed, fundamentally, at the entire nature-nurture framework [8, 41, 42]. My observations of Stephen, Susie and the other 8 children reinforce my insistence on understanding physical skill-making through a framework of developmental systems. In the beginning an adult-infant dyad contains the phenomenon. Gradually, the child separates from the dyad, but retains its developmental traces. Apportioning nature and nurture components in individual subjects is inappropriate; so too is a phenomenological approach, such as Iris Young’s, based solely on isolated adults.

To my knowledge only one other research group has offered longitudinal insight into learning to play with a ball [75]. This European lab (hence soccer!) watched the mother of a child named Beto, assigned male at birth, introduce him to a soccer ball at age 6 months. She demonstrated some of the ball’s properties (rolling and bouncing) and supported him under his arms so that his swinging legs “accidentally” kicked the ball. She and he played “kick-the-ball” in this fashion many times before he could walk. At 13 months he still needed his mother’s support but if she held only one hand he could manage to walk forward and give the ball a solid kick. At 22 months, in a moment developmental systems analysts would call a phase transition, he surprised both himself and his grandmother with a drop-kick ball return that made him leap in the air with pleasure. By three years and three months he had become more expert AND more verbal, and actively demonstrated to his brother how to kick. As Zukow-Goldring and colleagues wrote, “These richly textured interactions provide a window into the ‘‘hands on’’ social and perceptual processes…underlying how infants come to perceive, act, and know the organization and structure of everyday life.” (p. 580). Zukow-Goldring and Rader built a related framework for language development, which emphasized a continuous loop in the adult-infant dyad between perceiving and action [76].

The youngest infants we observed interacting with a ball were about 9 months old. Going forward in time, some of the babies became more adept at throwing, and engaged with their mothers in emotionally positive throwing games. Corbetta’s extensive work on learning to reach, provides a foundation for thinking about what precedes that moment when a baby reaches for, and grasps a ball. Starting at birth, a baby’s visual abilities and their spontaneous experiments with touch and motor proprioception barely connect. Until about four months depth perception and visual focus develop through experience and self stimulation. At the same time, but as a separate system, babies engage in ”an astonishingly high rate of spontaneous movements” and “touches to their body and the surrounding surface (>200 hand contacts over” a 10 min period).” [46] (p.420). During the first several months after birth, visual and touching actions connect and reinforce one another until a circular sequence of seeing, reaching, touching, grasping, manipulating and then, again, seeing, the result emerges. It is this last sequence—in this case grasping a ball—that I describe starting at about 9 months. (for an illustration, see Fig. 1, p. 410 in [46]).

### Conclusion: what about gender/sex?

What does all this have to do with gender/sex? What happens when Stephen and Susie begin, between 18 and 24 months to think “Huh? Gender?”, when they begin to express gender awareness? The starting points for “throwing like a boy or a girl” are already woven into their neuro-motor connections. Although they will each refine their skills and interests, the very fact that one began to play ball before he could walk and had many practice sessions and excited play with his mother, and the other learned to throw and walk at the same time (and had few practice sessions) has steered them along different pathways. As they link self-knowledge about their bodily skills with incoming information about ball-playing in the context of gender, it is a good guess that they also weave that strand into their own sense of identity.

This cascade of events probably started before birth. Stern (1985) speaks of a ghost in the nursery [77] (Kindle location 2063). This “haint” appears when an interaction between mother and child evokes a maternal embodied memory of interactions with *her* mother. It is fair to ask whether Stephen’s mother’s “instinct” to engage him with ball play, isn’t the nursery ghost at work. The same can be said for the mother who pushes a perfectly good ball aside so that she can encourage her child to “go shopping” with a toy shopping cart. In Adolph and Hoch’s terminology this grandparental gender ghost is one source for the enculturation of motor movements [69].

Here is my final provocation. If someone does NOT throw like a girl, does that mean they throw like a boy? Are throwing style and throwing force binary? I sincerely doubt it. If, as I urge in this paper, we follow development using longitudinal, naturalistic, qualitative observations, I suspect we will uncover a great deal of individual variation. Children will vary in the timing and method by which they acquire increasingly complex motor skills. Households and dyads will vary in the frequency with which ball play is an important feature of early development. And we will encounter training and feedback offered by peers and older siblings. If what truly exists is—dare I say it—a *spectrum* of throwing styles--we can, certainly, continue to use standard statistics to create binary gender/sex bins [78]. In doing so, however, we will lose insight into how motor skills develop in babies and how they are promoted and refined as children grow to adulthood. Instead, I urge using qualitative longitudinal, naturalistic observations to help frame specific questions aimed at investigating individual variation rather than aggregate group differences.

## Data Availability

No datasets were generated or analysed during the current study.

## Footnotes

1 Did they really mean sex? And if so, did they mean genitalia at birth? Or did they mean gender as a set of cultural practices, e.g. wearing dresses or pants, permission for free-range play, etc. A general fault of this entire literature is that it never carefully defines these words.

2 None of the names are the real names of these children.

3 In this last, outdoor session, Susie’s ball is a little bigger than Stephen’s which probably also affects the mechanics of lifting, but this was not the case in the first session.
